# Profiling and quantitative evaluation of three Nickel-Coated magnetic matrices for purification of recombinant proteins: lelpful hints for the optimized nanomagnetisable matrix preparation

**DOI:** 10.1186/1477-3155-9-31

**Published:** 2011-08-08

**Authors:** Mohammad Reza Nejadmoghaddam, Mahmood Chamankhah, Saeed Zarei, Amir Hassan Zarnani

**Affiliations:** 1 Nanobiotechnology Research Center (NBRC), Avicenna Research Institute, ACECR, Tehran, Iran; 2 Monoclonal Antibody Research Center (MARC), Avicenna Research Institute, ACECR, Tehran, Iran; 3 Immunology Research Center, Tehran University of Medical Sciences, Tehran, Iran

## Abstract

**Background:**

Several materials are available in the market that work on the principle of protein magnetic fishing by their histidine (His) tags. Little information is available on their performance and it is often quoted that greatly improved purification of histidine-tagged proteins from crude extracts could be achieved. While some commercial magnetic matrices could be used successfully for purification of several His-tagged proteins, there are some which have been proved to operate just for a few extent of His-tagged proteins. Here, we address quantitative evaluation of three commercially available Nickel nanomagnetic beads for purification of two His-tagged proteins expressed in *Escherichia coli *and present helpful hints for optimized purification of such proteins and preparation of nanomagnetisable matrices.

**Results:**

Marked differences in the performance of nanomagnetic matrices, principally on the basis of their specific binding capacity, recovery profile, the amount of imidazole needed for protein elution and the extent of target protein loss and purity were obtained. Based on the aforesaid criteria, one of these materials featured the best purification results (SiMAG/N-NTA/Nickel) for both proteins at the concentration of 4 mg/ml, while the other two (SiMAC-Nickel and SiMAG/CS-NTA/Nickel) did not work well with respect to specific binding capacity and recovery profile.

**Conclusions:**

Taken together, functionality of different types of nanomagnetic matrices vary considerably. This variability may not only be dependent upon the structure and surface chemistry of the matrix which in turn determine the affinity of interaction, but, is also influenced to a lesser extent by the physical properties of the protein itself. Although the results of the present study may not be fully applied for all nanomagnetic matrices, but provide a framework which could be used to profiling and quantitative evaluation of other magnetisable matrices and also provide helpful hints for those researchers facing same challenge.

## Background

After introduction of metal chelate affinity chromatography, a new approach to protein fractionation [1] and describing a new chelating matrix, Ni-NTA, for purification of fusion proteins containing histidine tags [2,3], His-tag affinity purification has been widely used for the purification of recombinant proteins from various expression systems [4-6]. In recent years, a broad array of common support matrices with slightly different materials, magnetic properties, adsorbent particle size and shape, and spatially binding capacities and strengths have been introduced as tricky reagents for successful purification process of His-tagged proteins [7,8].

With respect to these properties, the matrices offered by different commercial vendors differ very substantially from one another. Indeed, the choice of matrix is complicated by the fact that various suppliers offer practically the same particles under different names [7]. A collection of suppliers for nanomagnetic beads commonly used for the purpose of protein purification can be found in http://www.magneticmicrosphere.com/suppliers/magnetic_microspheres.php.

Meanwhile, designing a purification procedure employing magnetisable solid phase support has become one of the interesting issues among chromatography reagents for His-tagged protein purification due to their less susceptibility to sample viscosity, convenience for scaling up and automation [9-18]. In these research reports and also commercially available manuals, little information is available on their performance, and it is often quoted that greatly improved purification of histidine-tagged proteins from crude extracts could be achieved. Although these statements may be true in some cases, the lack of well-suited optimized purification protocol based on Nickel-coated magnetic matrices may lead variable or even contrasting results for purification of His-tagged proteins and presents a major limitation for broad application of such materials. In this regard, optimization and evaluation of commercially available matrices is mandatory, which may result in uniform purification efficacy. Performance of such commercial magnetic matrices for purification of different His-tagged proteins is, therefore, required to be evaluated in terms of specific binding capacity, percent yield and recovery and reproducibility. Although several helpful hints have been proposed to obtain good results in magnetic separations of proteins and peptides [15], the full potential of these techniques has not been fully exploited. The present paper describes the evaluation and optimization of three newly-released magnetic beads namely: SiMAC-Nickel, SiMAG/N-NTA/Nickel and SiMAG/CS-NTA/Nickel for purification of two His-tagged recombinant proteins, His-ProT and His-Mre11, overexpressed in *Escherichia coli*.

## Results

### Relative expression of His-tagged recombinant proteins

**Figure 1:**
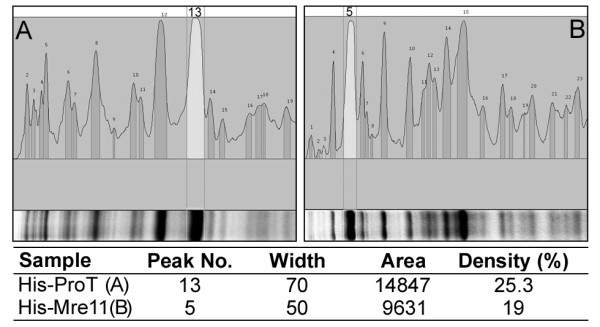
**Densitometric analysis of recombinant protein expression**. ProT (A) and Mre11 (B) recombinant proteins were expressed in ***E.coli ***and their relative expression in the soluble fraction of cell lysate were determined by densitometry using AlphaEase software.

Recombinant proteins were extracted from IPTG-induced bacteria and their expression rates in the soluble fractions of cell lysate were determined by densitometric analysis as the percent of specific band to the all bands observed in SDS-PAGE gel. Accordingly, ProT and Mre11 relative expression rates were estimated to be about 25 and 19 percent, respectively (Figure 1).

### Evaluation of beads specific binding capacity

**Figure 2:**
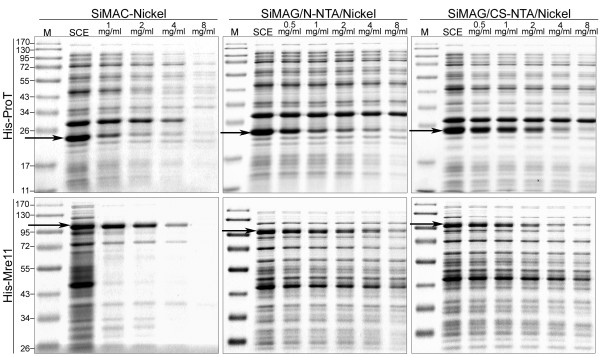
**SDS-PAGE analysis of flowthrough fractions of His-recombinant proteins bound onto the different concentrations of three Nickel-coated magnetic matrices**. His-ProT and His-Mre11 recombinant proteins in soluble cell extract (SCE) of ***E.coli ***were bound to increasing concentrations of magnetic matrices, SiMAC-Nickel, SiMAG/N-NTA/Nickel and SiMAG/CS-NTA/Nickel, and flowthrough fraction of each matrix at each concentration was subjected to SDS-PAGE analysis. The target proteins are shown by black arrows.

**Figure 3:**
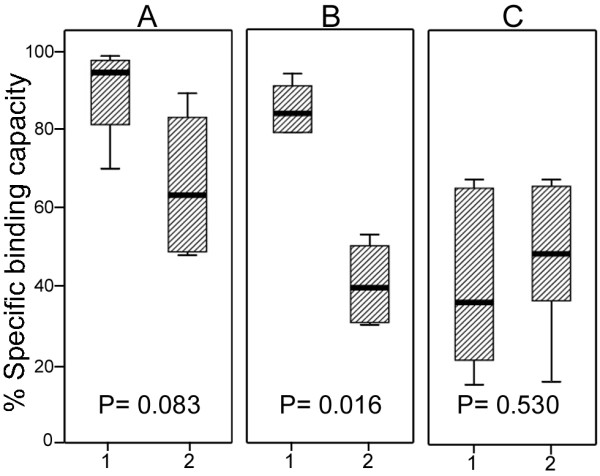
**Specific binding capacity of three Nickel-magnetic matrices for two His-tagged recombinant proteins**. After binding of His-tagged recombinant proteins, His-ProT and His-Mre11, onto the Nickel-magnetic matrices, SiMAC-Nickel, SiMAG/N-NTA/Nickel and SiMAG/CS-NTA/Nickel, flowthrough fractions (FT) were subjected to SDS-PAGE analysis. Percent of band density in FT subtracted from 100% was defined as specific binding capacity. His-ProT (1), His-Mre11 (2), SiMAC-Nickel (A), SiMAG/N-NTA/Nickel (B) and SiMAG/CS-NTA/Nickel (C).

At first, according to recommendation of the manufacturer, purification of His-tagged proteins was carried out based on the protocol supplied by Frenzel et al. [11] with 70 mg/ml of the beads and final elution of purified protein by 0.25 M imidazole solution. By applying this protocol, most of the His-proteins remained attached to the beads after elution (data not shown) and this prompted us to look for an optimized procedure to purify His-tagged proteins. The effect of the different magnetic beads concentrations (from 1 to 8 mg/ml for SiMAC-Nickel and from 0.5 to 8 mg/ml for SiMAG/N-NTA/Nickel and SiMAG/CS-NTA/Nickel) on His-ProT and His-Mre11 specific binding capacity at pH 8.0, 4°C was investigated by measurement of relative density of specific band in flowthrough (FT) fractions (Figure 2). In the range of bead concentration examined, maximum target proteins binding capacity was achieved at concentration of 8 mg/ml for all magnetic matrices examined (Figure 2 and Table 1). As shown in Figure 2, besides to proteins of interest, a number of non-target proteins was adsorbed non-specifically to SiMAC-Nickel beads and demonstrated a very similar trend of adsorption with increasing the concentration of the bead. As with His-tagged proteins, total content of non-specific proteins in FT decreased with increasing the concentration of SiMAC-Nickel beads indicating non-specific binding of non-target proteins in parallel to the target proteins. This pattern was not observed in the other two magnetic beads (Figure 2), where, content of target proteins in FT decreased considerably by increasing the concentration of the beads, whereas that of the contaminating proteins remained unchanged. Densitometric analysis of FT fractions revealed that three magnetic beads have different biding capacity and behave differentially as far as different His-tagged proteins are concerned. While SiMAC-Nickel and SiMAG/CS-NTA/Nickel specifically bound to both His-ProT and His-Mre11 proteins at comparable levels, the binding capacity of SiMAG/N-NTA/Nickel beads to His-ProT was significantly greater than His-Mre11(Figure 3) (p = 0.016)

**Table 1:** Purification efficacy records of three Nickel-coated magnetic matrices for His-ProT and His-Mre11 recombinant proteins

Resin type	Protein type	Bead Concentration (mg/ml)	Specific binding capacity (%)	Relative band density (%)				Yield (%)	Recovery (%)	Loss (%)
							
				E_1_	E_2_	E_3_	E_4_			
SiMAC-Nickel	His-ProT	1	68.1	11.9	10.5	10.2	10.5	43.1	63	25
		2	91.5	12.6	16.3	21.7	27.8	78.4	86	13.1
		4	95.7	1.8	3.8	11.6	64.5	81.7	85	14
		8	98	0.5	0.6	1.1	66.2	68.4	70	29.6
	
	His Mre11	1	45.1	2	0.1	0	0.1	2.2	5	42.9
		2	46.9	2.1	0.1	0	0.1	2.3	5	44.6
		4	75.3	3	0.4	0	0.1	3.5	5	71.8
		8	88.1	3.6	0.9	0	0.1	4.6	5	83.5

SiMAG/N-NTA/Nickel	His-ProT	0.5	50.4	17.8	12.8	1.6	0.4	32.6	65	17.8
		1	77.7	41.5	23.9	3.6	0.3	69.3	89	8.4
		2	82.8	29.2	37.8	9.4	1.1	77.5	93	5.3
		4	90.1	11.8	39.2	28.3	5.4	84.7	94	5.4
		8	93.3	6.5	34.2	32	7.7	80.4	86	12.9
	
	His Mre11	0.5	26.6	15.7	1.3	0.6	0.3	17.9	67	8.7
		1	27.2	16.3	1.2	1.1	0.7	19.3	71	7.9
		2	36.4	1.3	11.4	13.6	1.2	27.5	75	8.9
		4	47.6	1.1	12.9	15.8	6.5	36.3	76	11.3
		8	50.6	0.9	2.3	10.9	5.5	19.6	38	31

SiMAG/CS-NTA/Nickel	His-ProT	0.5	11.2	0	0	0	0	0	0	11.2
		1	17.7	3.4	0.1	0.1	0.1	3.7	21	14
		2	33	5.2	3.1	1.6	1.8	11.7	35	21.3
		4	63.4	8.6	19.1	9.2	2.5	39.4	62	24
		8	65.7	12.8	14.9	3.9	1.9	33.5	51	32.2
	
	His Mre11	0.5	12	0.5	1	1.2	4.1	6.8	57	5.2
		1	33.5	0.8	1.2	8.9	13.5	24.4	73	9.1
		2	46	4.1	2.6	11.7	18.5	36.9	80	9.1
		4	63.9	4.1	2.6	11.7	27.4	45.8	72	18.1
		8	65.7	4.1	2.6	11.7	27.3	45.7	69	20

E_1_-E_4 _are imidazole concentration for elution of recombinant proteins ranging from 0.25, 0.5, 1 to 2 and 0.05, 0.1, 0.25 to 0.5 molar (M), For SiMAC-Nickel beads and SiMAG/N-NTA/Nickel or SiMAG/CS-NTA/Nickel, respectively. Specific binding capacity: Percent of band density in flowthrough fraction (FT) for each bead concentration subtracted from 100% was defined as Specific binding capacity. Yield: was defined as the sum of the percents of the specific band densities at four elution steps (E1-4). Recovery: was calculated as the percent of purification yield divided by Specific binding capacity. Protein loss: The sum of the percents of specific band densities in four wash steps (W1-4) and residual fraction (RF) was defined as protein loss.

### Protein yield and recovery

**Figure 4:**
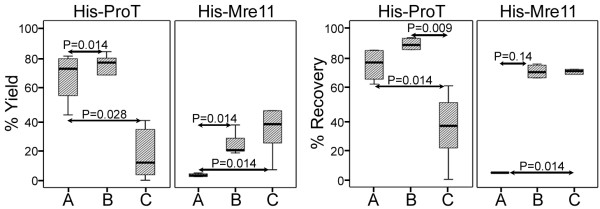
**Comparison of purification yield and protein recovery of three Nickel-magnetic matrices for His-ProT and His-Mre11 recombinant proteins**. Purification yield was defined as the sum of the percents of the specific band densities at four elution steps (E1-4). Recovery percent was calculated as the percent of purification yield divided by specific binding capacity. SiMAC-Nickel (A), SiMAG/N-NTA/Nickel (B) and SiMAG/CS-NTA/Nickel (C).

**Figure 5:**
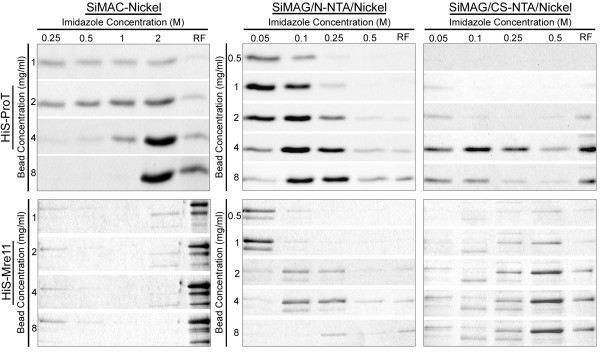
**Effect of imidazole concentration on elution of recombinant proteins from three Nickel magnetic matrices**. His-ProT and His-Mre11 recombinant proteins were bound onto the different concentrations of SiMAC-Nickel, SiMAG/N-NTA/Nickel and SiMAG/CS-NTA/Nickel magnetic matrices. Elution fractions collected by increasing concentrations of imidazole were subjected to SDS-PAGE analysis. RF: Residual fraction.

**Figure 6:**
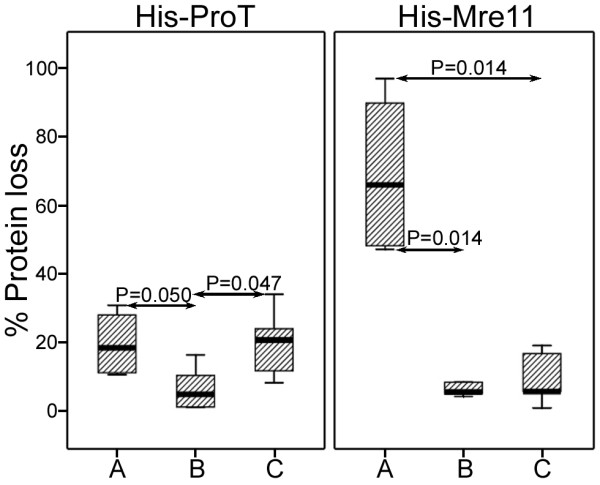
**Percent loss of target recombinant proteins purified by three Nickel magnetic matrices**. Percent of the recombinant proteins, His-ProT and His-Mre11, lost during purification process by SiMAC-Nickel, SiMAG/N-NTA/Nickel and SiMAG/CS-NTA/Nickel magnetic matrices was calculated as described in materials and methods. Comparison was made between three matrices for each protein. SiMAC-Nickel (A), SiMAG/N-NTA/Nickel (B) and SiMAG/CS-NTA/Nickel (C).

In order to compare the efficacy of three magnetic/Nickel beads in protein purification, two further indices were evaluated. Yield and recovery percents were calculated as mentioned in methods. Interestingly, three matrices showed completely different purification efficacy as far as such variables as bead concentration, imidazole concentration, and the type of His-tagged protein were concerned (Table 1). The best purification result in terms of both yield and recovery percent was obtained for His-ProT when it was purified by 4 mg/ml of SiMAG/N-NTA/Nickel beads (Table 1 and Figure 4). The least efficacy of His-ProT purification was observed with SiMAG/CS-NTA/Nickel beads where a considerable amount of protein did not elute after four elution steps (Figure 4). Indeed, in comparison to other beads, SiMAG/CS-NTA/Nickel bead did not show reasonable specific binding capacity to this protein (Table 1 and Figure 4). These elution patterns were different from those of His-Mre11 protein, in which His-Mre11 protein was not purified at all by SiMAC-Nickel beads (Table 1 and Figure 5). In this case, approximately all bound proteins remained attached to the matrix even after elution with 2 M concentration of imidazole (Table 1). Protein loss was considerably higher when His-Mre11 was purified by SiMAC-Nickel bead compared to the other beads (Figure 6) (P = 0.014). Although, the highest recovery and yield for His-Mre11 were obtained when it was purified by 4 mg/ml of SiMAG/CS-NTA/Nickel bead (Table 1), the presence of nonspecific bands during the elution steps as judged by SDS-PADE (Figure 5) render it unsuitable for protein purification. Regarding the total protein loss for both proteins (Table 1), the SiMAG/N-NTA/Nickel bead was superior to the other beads.

### Effect of imidazole concentration

According to the methods, proteins were eluted from SiMAC-Nickel beads by increasing concentrations of imidazole solution starting from 0.25 M and continued till 2 M. Our preliminary data showed that neither His-Mre11 nor His-ProT is eluted by lower concentrations of imidazole (data not shown). This condition was in contrast to what we observed in SiMAG/N-NTA/Nickel or SiMAG/CS-NTA/Nickel beads where elution was taken place with as low as 0.05 M of imidazole solution. In this context, using SiMAG/N-NTA/Nickel bead, His-ProT was eluted the most by 0.1 and 0.25 M imidazole solution, while it remained attached to the SiMAC-Nickel bead until higher concentration of imidazole (2 M) was used (Table 1). The results of the elution experiments with different concentrations of imidazole have been summarized in Table 1 and shown in Figure 5. As expected, the higher the concentration of beads, the higher fraction of the protein remained attached to the matrix (Figure 5).

### Effect of bead concentration

In order to clarify the effect of bead concentration on the purification efficacy, different concentrations of beads were examined. As shown in Table 1, specific binding capacity of the beads for both recombinant proteins was increased considerably by increasing their concentrations. Moreover, in the case of SiMAC-Nickel there was a direct relationship between the bead concentration and the concentration of imidazole solution required for protein elution (Figure 5). More importantly, the higher the bead concentration, the more protein remained uneluted even after the application of the highest concentration of elution buffer (Table 1 and Figure 5). Furthermore, the purity analysis of eluted proteins by SDS-PAGE and subsequent silver staining showed that at bead concentrations greater than 4 mg/ml several contaminating proteins were present in addition to target His-tagged protein. This analysis showed that usage of lower concentration of the beads during binding process may reduce relative percentage of non-specific protein adsorption and thereby increases the purity. Nevertheless, when the bead concentration was further decreased, the purification yield was decreased in parallel.

4 mg/ml of SiMAG/N-NTA/Nickel bead resulted in the best purification result in terms of both yield and recovery for His-ProT. The same concentration of SiMAC-Nickel bead was efficient for purification of His-ProT as well, but higher concentrations of imidazole were needed the protein to be recovered (Table 1 and Figure 5).

### Verifying the purified His-tagged Proteins by Western blotting

**Figure 7:**
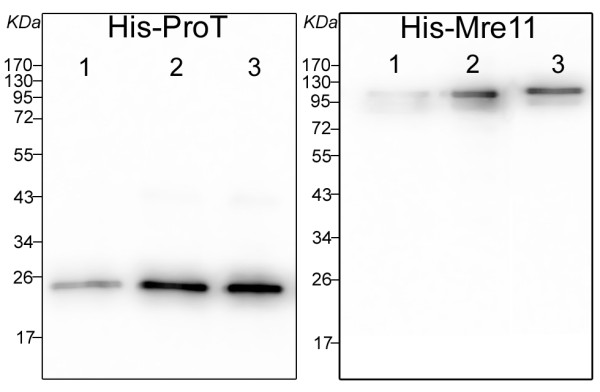
**Western blot analysis of purified His-ProT and His-Mre11 recombinant proteins**. Elution fractions of His-ProT and His-Mre11recombinant proteins purified by 4 mg/ml SiMAG/N-NTA/Nickel magnetic matrix were subjected to SDS-PAGE. Bands were transferred to nitrocellulose membrane and specific bands were detected by antibodies directed against 6His tag by ECL system. 1-3 indicated the fractions eluted by 0.05, 0.1 and 0.25 M imidazole, respectively.

The recombinants His-ProT and His-Mre11 in the eluate had molecular masses of about 20 and 100 kDa, respectively, when analyzed by SDS-PAGE. As representative for all matrices, purified proteins from SiMAG/N-NTA/Nickel beads were also characterized using specific antibodies by Western blotting which showed the expected bands as depicted in Figure 7.

## Discussion

Magnetic-based His-tag affinity matrices have been widely used for the purification of recombinant proteins from various overexpression systems [4,5,15]. Given their wide application in protein purification, setting the optimal conditions up to achieve the best recovery, yield and purity covering the wide range of recombinant proteins is a prerequisite. In most instances, however, general procedures are usually described, not pointing to the details of methodology in terms of optimal matrix: lysate ratio, elution conditions, purification quality or final yield. This is mostly true for newly-released commercial matrices which are not supported by the existing data in the literature. Although, it is believed that the purity and yield of such procedures depend to some extend on the protein itself [4,11], evaluation of the procedure itself deserve to be performed extensively. The present study evaluated three new commercial magnetic matrices quantitatively and qualitatively and compared their efficacy for purification of the two recombinant His-tagged proteins, ProT and Mre11.

Our observations showed that these matrices give considerably different purity, yield, and have different specific binding capacity and recovery. Evaluation of flowthrough fractions clearly showed that besides protein of interest, SiMAC-Nickel matrix adsorbs unrelated proteins as well from the expression system. It is notable that SiMAC-Nickel matrices are porous in nature, a character which may explain their extra ordinary non-specific adsorptive capacity for irrelevant proteins. In line with this finding, Franzerb et al. [7] proposed that matrix should be non-porous with respect to the target biomolecules. On the other hands, this matrix is consisted of a magnetic core and a nickel-silica composite matrix with the nickel ions tightly integrated in the silica [11] and so, in contrast to NTA-coupled matrices, all valences of Ni are available for histidine binding. This may result in increased binding to His-like endogenous proteins as impurities. Thus, it seems that the surface chemistry of the matrix is an important determinant which affects the degree of non-specific interactions. Indeed, the percent of non-specific binding was not only influenced by the type of the matrix, but apparently depended on the nature of the His-tagged protein as well (See Figure 5). We encountered minimal problem with purification of His-ProT and in this case the impurities were minimal as well, but with Mre-11, which is a high MW protein, not only the purification efficacy was low, but there was a considerable amount of non-specific proteins eluted in conjunction with this protein. Final purity of the purified proteins is without any doubt an excellent measure of the performance of protein purification systems. In this regard, SiMAG/N-NTA/Nickel showed superior quality over the SiMAG/CS-NTA/Nickel. Specific binding performance of the matrixes for ProT and Mre11 also showed great variation. This is mainly influenced by the type of the matrix. One determining factor which affects both specific banding capacity, % yield and recovery is the affinity of interaction between matrix and the protein of interest which in turn is determined by the number of coordination bands available in the matrix. According to the information provided by the manufacturer, SiMAG/CS-NTA, and SiMAG/N-NTA are synthesized by a one-step coupling procedure of Nitrilotriacetic acid (NTA) to SiMAG-Carboxyl via EDC [1-Ethyl-3-(3-dimethylaminopropyl) carbodiimid] activation. The difference between SiMAG/N-NTA/Nickel and SiMAG/CS-NTA/Nickel is caused in part by a different carboxylation degree of the starting material; SiMAG-Carboxyl. NTA adsorbents including SiMAG/CS-NTA and SiMAG/N-NTA are quadridentate chelate former and form four coordination bands with such metal ions as Nickel. Regarding the fact that Ni has six valencies, only two valences remain unoccupied for reversible binding to histidine [3]. This may explain the higher affinity and binding capacity of SiMAC-Nickel, which has six coordination bounds available for histidine binding, compared to the other two NTA-based matrices.

Collectively, SiMAG/CS-NTA/Nickel showed lower specific binding capacity compared to the other beads. Such limitation should be overcome if the costs of recombinant protein production are to be lowered.

As a matter of fact, a purification system should give as high yield as possible with high recovery and could be applicable to a broad range of proteins. A purification system working well only on a specific group of proteins could not be desirable. In this context, SiMAC-Nickel matrices were inferior to both SiMAG/N-NTA/Nickel and SiMAG/CS-NTA/Nickel matrices because it was unable to recover the majority of Mre11. Although, both Mre11 and ProT were recovered by SiMAG/N-NTA/Nickel and SiMAG/CS-NTA/Nickel beads, SiMAG/N-NTA exhibited superior capacity when % recovery for both proteins was concerned. Three matrices also showed variable yields with similar pattern as recovery. As a whole, SiMAG/N-NTA/Nickel bead was superior in terms of both yield and recovery regardless of the type of protein.

Another important factor which should be taken in mind for all protein purification systems is the strength needed for elution of the proteins from matrix. The harsher the elution condition, the more likely protein loses its structure and function. Our data showed that higher concentration of imidazole is needed the proteins to be eluted from SiMAC-Nickel beads. This was in contrast with the elution pattern of SiMAG/N-NTA/Nickel matrices in which lower concentrations of imidazole were quite sufficient for proteins elution. These differences can be attributed to the higher affinity of SiMAC-Nickel beads to the His-tagged proteins compared to the NTA-coupled matrices. Therefore, on the view of elution conditions, SiMAG/N-NTA/Nickel matrices were superior as well.

In contrast to what has been reported earlier [11], our results showed that higher concentration of the matrix, binding more His-tagged proteins doesn't usually lead to the best yield and purification results. This conclusion was supported by the fact that higher concentrations of imidazole, which can disrupt macromolecular complexes, were required to elute out the majority of His-tagged proteins from the beads when higher concentrations of the beads were used (more than 4 mg/ml). At this high bead concentration, a fraction of His-tagged protein was still remained bound to the matrices after multiple imidazole elutions which resulted in lower yield. The reason for this notion is that with higher bead concentrations, higher Ni ions would be accessible to interact with histidine moieties on recombinant protein which in turn strengthen the affinity of interaction. This may lead the His-tagged protein to be remained bound to the beads after elution step [19]. Indeed, at higher bead concentrations non-target proteins (including His-tag like endogenous host and hydrophobic proteins which bind to Ni ions and matrix of beads, respectively) contaminated the protein of interest in the eluate.

As a result, application of optimal bead concentration during protein binding (here 4 mg/ml) may not only increases the purity of target protein by leaving fewer opportunities for both His-tag like endogenous and other non-specific host proteins to be bound onto the nickel ions and matrix itself, respectively, but may improve the quality of purified recombinant protein by allowing lower concentrations of imidazole to be used for elution. It should be noted that when the bead concentration is further decreased, His-tagged proteins are lost during wash steps.

Therefore, it should be taken in mind that purification indices are completely interrelated with positive and negative impacts on each other and a compromise should be made for selection of the best purification system. Taken together, we conclude that SiMAG/N-NTA/Nickel would be the matrix of choice to get uniform results for different His-tagged proteins.

Until now several helpful hints have been proposed to obtain good results in magnetic separations of proteins and peptides [15]. The provided information in this report could be viewed as a clue helping researchers to overcome obstacles raised during purification of His-tagged recombinant proteins by Nickel-coated magnetisable matrices.

## Conclusions

Protein purification using magnetisable solid phase supports have still been accompanied by some fundamental drawbacks. The extent of specific binding capacity, purity, yield and recovery vary from one matrix to another. This variability is a function of structure and surface chemistry of the matrix which are determining factors for affinity of interaction. It is also influenced to a lesser extent by the physical properties of the protein, itself. The present paper represents a reliable methodology for assessment of functionality of different nanomagnetic matrices working with the same principle. And more importantly, points to step by step optimization procedure for purification of His-tagged recombinant proteins. Although the results of the present study may not be fully applied for all nanomagnetic matrices, but provide a framework which could be used to profiling and quantitative evaluation of other magnetisable matrices, especially those useable for His-tagged protein purification. The final goal is, without any doubt, manufacturing a versatile nanomagnetic matrix and introducing an optimized protocol functioning over a majority of recombinant proteins. In this context, devoting further research efforts on production and optimizing of such nanomagnetisable matrices is a necessity which would help to give new insights for developing versatile and user-friendly resins suitable for purification of a vast array of recombinant His-tagged proteins.

## Methods

### Instruments

Magnetic separation stand and permanent magnet separator were purchased from Promega company (Madison, WI USA). Other major instruments used in this study were: GFL 3033 (Burgwedel, Germany) and SHEL Lab (Oregon, USA) shaking incubators for bacterial culture and recombinant protein expression, Sonoplus HD 2070 sonicator (Bandelin, Berlin, Germany) for bacterial cell lysis, UV/Visible Biophotometer (Ependorf, Hamburg, Germany) for Bradford assay, Eppendorf 5810R and 5415R refrigerated centrifuges, and Bio-Rad electerophoresis system for sodium dodecylsulfate-polyacrylamide gel electrophoresis (SDS-PAGE) (Bio-Rad Laboratories, California, USA).

### Chemicals

New versions of the Nickel-Magnetic beads: SiMAC-Nickel, SiMAG/N-NTA/Nickel and SiMAG/CS-NTA/Nickel were purchased from Chemicell company (Berlin, Germany) (Table 2). Chemicals used were of molecular biology grade. DTT, TEMED, Acrylamide/bis-acrylamide and PMSF were purchased from Sigma (St Louis, Mo., U.S.A). The expression vector pET19b and E. coli strain BL21 (DE3) were purchased from New England BioLabs (Ontario, Canada), DNase I and RNase A were from Roche applied science (Penzberg, Germany). Isopropyl ß-thiogalactopyranoside (IPTG) was from Gibco (Gaithersburg, MD, USA). Imidazole was from USB (Cleveland, OH, USA). The prestained protein ladder consisting of different arrays of molecular weights 170, 130, 95, 72, 56, 43, 34, 26, 17 and 11 kDa was from Fermentas (St. Leon-Rot, Germany). Reagents for Bradford protein assay were purchased from Bio-Rad Laboratories (Bio-Rad Laboratories, California, USA). All other chemicals were from Sigma-Aldrich unless otherwise stated.

**Table 2:** Characteristics of magnetic nanomatrices used in this study

Beads Name	Concentration	Functional group group
SiMAC-Nickel	100 mg/ml	Silica-nickel
SiMAG/N-NTA/Nickel	50 mg/ml	NTA- nickel
SiMAG/CS-NTA/Nickel	50 mg/ml	NTA- nickel

### Recombinant Proteins to be purified

Two different recombinant proteins with six histidine residues (His-tag) in their C-terminus, ProT and Mre11, with molecular weights of about 25 and 100 KD, respectively, were chosen to be separated using the Nickel-coated magnetic beads. Both proteins were expressed in to the bacterial cytosol.

### Growth of bacteria and induction of gene expression

The expression plasmids, pET19b/Mre11 and pET19b/ProT were prepared and transformed into E. coli BL21 (DE3) as host strain. The Mre11, is a central part of a multisubunit nuclease composed of Mre11, Rad50 and Nbs1 (MRN) [20]. The MRN complex plays a critical role in sensing, processing and repairing DNA double strand breaks [21]. Three millilitres of SOB medium [5.0 g tryptone, 1.25 g yeast Extract, 0.125 g NaCl, 0.0465 g KCl per 250 ml water, pH 7.0 containing ampicillin (100 μg/ml)] were inoculated with a single colony of the transformed BL21(DE3) and grown overnight at 37°C with shaking at 225 rpm. The next day, 12 ml of prewarmed SOB medium were inoculated with the overnight culture medium until the final OD600 nm was reached to 0.1 [having the OD600 nm of about 4-5, 250 μl of the overnight culture in 12 ml of fresh SOB medium gave an OD of 0.1]. The culture was grown at 37°C with shaking at 225 rpm to an OD600 nm of 0.4-0.5. At this point, protein expression was induced by 12 μl of 1 M IPTG to give a final concentration of 1 mM. The induced culture was continued for 4 hours and then processed for protein extraction. During the expression processes, a sample of 250 μl was taken at the end of each hour for SDS-PAGE analysis.

### Cell lysis and protein extraction

Bacterial cells were harvested by centrifugation of cell culture at 4000 rpm, 4°C for 10 min. Supernatant was aspirated off and cells were washed three times with cold binding-wash solution (20 mM Na_2_HPO_4_, pH 7.0). Cells were then resuspended in 2 mL cold lysis buffer (20 mM Na_2_HPO_4_, 10 mM imidazole, pH 7.0, 1 mM PMSF, and 27 mM lysozyme) and incubated on ice for 30 minutes. Cell lysis was further continued by sonication (10 s at 70% power, four times, 1 min intervals at 4°C with a M73 probe). The lysate was centrifuged at 12000 rpm, 4°C for 10 min and 1 ml of supernatant was transferred into a 1.5-mL eppendorf tube. At the next step, RNase A and DNase I (0.125 μg/ml and 3 Unit/ml final concentrations, respectively) were added and incubation was continued on ice for 10-15 minutes. After centrifugation at 13000 rpm for 10 min, 4°C, supernatant was filtered through a 0.2 μm cellulose acetate filter (Millipore, USA) before mixing with Nickel-coated magnetic beads.

### Estimation of total protein concentration

The protein concentration of filtered soluble cell extract (SCE) was estimated by spectrophotometric analysis at 280 nm in an UV/Visible biophotometer and confirmed by Bradford assay [22] using bovine serum albumin as standard.

### Protein Purification by Nickel-coated magnetic beads

Different amounts of Nickel-coated magnetic beads [5, 10, 20 and 40 μl of SiMAC-Nickel bead (100 mg/ml) corresponding to the final concentration of 1, 2, 4 and 8 mg/ml, respectively, and 5, 10, 20, 40 and 80 μl of SiMAG/N-NTA/Nickel and SiMAG/CS-NTA/Nickel beads (50 mg/ml) corresponding to the final concentration of 0.5, 1, 2, 4 and 8 mg/ml, respectively] were transferred to eppendorf tubes. Tubes were placed on a magnet until the beads migrated to the side of the tube and the clarified liquids were discarded. The beads were washed and equilibrated three times with 500 μl of cold lysis buffer. Meantime, soluble cell extracts were diluted to a final concentration of 1.5 mg/ml with cold lysis buffer before mixing with beads. Diluted SCE was added to the beads in final volume of 700 μl. The mixture mixed well by gentle pipetting and incubated for 30 minutes on a roller mixer (Behdad Roller Mixer, Tehran, Iran) at 4°C for protein binding. After the binding process, tubes were placed in the magnetic separator, and except a small volume (30 μl) of the clarified supernatant which was collected and frozen for further analysis as flowthrough samples (FT); the rest was removed and discard. Wash steps were performed 4 times by adding 500 μl of wash buffer (50 mM NaH_2_PO_4_, 300 mM NaCl, 10 mM imidazole, pH 8.0), gentle pipitting and mixing on a roller mixer each for 5 min. At each washing steps, a small portion of supernatant was collected (W1-4) and the rest was discarded. After four washing steps, the entrapped His-tagged proteins were eluted with 200 μl of elution buffers (50 mM NaH_2_PO_4_, 300 mM NaCl containing different concentrations of imidazole 250 mM, 500 mM, 1 M or 2 M imidazole, pH 8.0 for SiMAC-Nickel bead and 50 mM, 100 mM, 250 mM and 500 mM imidazole for SiMAG/N-NTA/Nickel and SiMAG/CS-NTA/Nickel beads). Briefly, 200 μl of elution buffer was added to the beads and mixed as above. After magnetic separation, the clarified liquid containing the eluted His-tagged protein were transferred into microtubes followed by centrifugation at 12000 rpm for 3 minutes. Supernatant from each elution steps (E1-4) was then collected and stored at - 20°C. To evaluate the elution efficacy, the beads pellet was admixed with 500 μl of 1× SDS-PAGE loading buffer (50 mM Tris-HCl pH 6.8, 10% glycerol, 2.5% SDS, 0.1% bromophenol blue, 25 mM Dithiothreitol), boiled for 5 min and subjected to SDS-PAGE as residual fraction (RF).

### SDS- PAGE and Western blotting

SDS-PAGE analysis was performed based on Laëmmli protocol [23]. Samples [soluble cell extract (SCE), flowthrough (FT), washes (W1-4) and elutions (E1-4)] were prepared by mixing 30 μl aliquots of each preparation with 7 μl of 5× loading buffer. The samples were boiled for three minutes and spinning down. Then 30 μl of supernatants in conjunction with 30 μl of residual fractions were loaded on 10-12% polyacrylamide gel. In case of *E.coli *cultures for recombinant protein expression, samples of 250 μl were collected during different intervals of induction process, centrifuged and the pellets were directly suspended in 150 μl of 5× loading buffer, shacked vigorously and then processed as above. Prestained protein ladder was used as molecular weight marker. Electrophoresis was performed in a Mini-Protean II apparatus (Bio-Rad Laboratories, Hercules, CA, USA) with running buffer composed of 25 mM Tris-HCl pH 8.3, 192 mM glycine, 0.1% SDS. After separation, gels were stained with silver nitrate. Western blot analysis was carried out according to the protocol we published elsewhere [24] with some modifications. Briefly, after transfer onto nitrocellulose membranes, blocking was done overnight in 5% skimmed milk followed by three washes with TBS-TT (20 mM Tris base, 500 mM NaCl, 0.1% v/v Tween 20, 0.4% v/v Triton x100 PH, 7.5), each for 10 min. Goat anti-His6 monoclonal antibody (Invitrogen, California, USA) and rabbit anti-Mre11 and anti-ProT polyclonal antibodies (Produced in our laboratory) were applied to the membrane at 1:3000 as primary antibody for 1.5 h followed by 1:3000 dilution of hoarse-radish peroxidase (HRP)-conjugated rabbit anti-goat or sheep anti-rabbit (Avicenna Research Institute, Tehran, Iran) for 1 h. Membrane was then washed as above and specific bands were developed by enhanced chemiluminiscent (ECL) system (GH Healthcare, Buckinghamshire, UK) according to the manufacturer's instruction using X-ray film processor (HOPE Micro-Max, Warminster, USA).

### Densitometric analysis

Silver-stained SDS-PAGE gels were scanned and density of specific bands for two recombinant proteins from samples collected at different purification steps (FT, W1-4, E1-4 and residual fraction) in five separate experiments was analyzed using the program AlphaEase FC Software (Version 5.0.1) with standard settings. The method of densitometry we employed was based on calculation of AUC (area under curve) which is based on both band density (height of the curve) and band area (width of the curve). This integrated density value normally offsets the possible mistakes which may be encountered when only band density is concerned. For each individual purification, the sum of the specific band densities from aforesaid fractions was set to 100% and relative percent of each band was calculated accordingly. The expression rate of each recombinant protein in the soluble fraction of cell lysate was determined by densitometric analysis as the percent of specific band to the all bands observed in SDS-PAGE gel.

### Determination of protein purification efficacy

Four indices including specific binding capacity, purification yield, and percent of protein recovery and loss were determined for each Nickel-magnetic matrix, each bead concentration and each recombinant protein. The sum of the specific band densities from FT, W1-4, E1-4 and RF were set to 100%. Percent of band density in FT subtracted from 100% was defined as specific binding capacity. Purification yield was defined as the sum of the percents of the specific band densities at four elution steps (E1-4). Recovery percent was calculated as the percent of purification yield divided by specific binding capacity. The sum of the percents of specific band densities in W1-4 and RF was defined as protein loss.

### Statistical Analysis

Numerical data analysis was done using SPSS software version 13.0 (SPSS Inc., Chicago, Illinois). Two-tailed statistical analyses were performed using the SPSS software version 13.0. Percent of bound, lost and eluted fractions of each protein was calculated for five individual experiments for each matrix and compared by Mann-Whitney test with Bonferroni correction. P-values less than 0.05 were considered significant.

## Competing interests

The authors declare that they have no competing interests.

## Authors' contributions

The authors meet the criteria for authorship as follows:

MRN has made substantial contribution to design, acquisition of data and manuscript drafting. MC has made substantial contribution to conception and design. SZ has participated in data analysis and AHZ has involved in methodology design, interpretation of data, critical revision of the manuscript and final approval of the version to be published.
